# Phenotypic and Cellular Characteristics of a Stromal Vascular Fraction/Extracellular Matrix Gel Prepared Using Mechanical Shear Force on Human Fat

**DOI:** 10.3389/fbioe.2021.638415

**Published:** 2021-02-26

**Authors:** Yuan Ye, Jingjiang Zou, Meijun Tan, Kuikui Hu, Jindou Jiang

**Affiliations:** Department of Plastic and Cosmetic Surgery, Guangdong Women and Children Hospital, Guangzhou, China

**Keywords:** SVF/ECM gel, Coleman fat, shear force, tissue regeneration, stem cells, flow cytometry

## Abstract

The retention of fat-derived grafts remains a challenge for regenerative medicine. Fat aspirates from patients undergoing liposuction were prepared into standard Coleman fat grafts or further isolated using mechanical shear force to prepare a stromal vascular fraction (SVF)/extracellular matrix (ECM) gel. The retention rate of the SVF/ECM gel was significantly higher than that of the Coleman fat at 3, 14, 28, and 60 days following transplantation on the backs of nude mice. The viscosity of the fat was directly proportional to the shearing force. Although the mechanical isolation did not affect the total number of cells, it significantly decreased the number of living cells. Flow cytometry showed a greater number of mesenchymal stem cells, supra-adventitial (SA)-adipose stromal cells (ASCs), and adipose-derived stem cells but a lower number of endothelial progenitor cells in the SVF/ECM gel than in the Coleman fat. Thus, mechanical isolation of fat can increase the pluripotency of adipocytes, which can improve graft retention in cell therapy.

## Introduction

Fat grafting is currently considered the standard procedure for repairing soft-tissue defects, which has been widely used in the fields of plastic, reconstructive, and esthetic surgery (Cohen et al., 2017; Rohrich et al., 2018; Cansancao et al., 2019; Li X. et al., 2019). However, the instability of long-term effects after fat transplantation limits the clinical application of fat grafting (Qiu et al., 2016). The success of fat transplantation is affected by several factors such as donor age, donor body mass index, donor site, and the methods of fat treatment and transplantation (Cucchiani and Corrales, 2016; Tuin et al., 2016). Currently, the fat treatment method is considered to be the most important factor influencing transplantation success (Canizares et al., 2017; Streit et al., 2017). Accordingly, the optimization of fat transplantation has attracted increasing attention in the field of regenerative medicine. Novel fat transplantation schemes have been developed over the last decade to minimize intervention, with the goal of destroying several mature adipocytes while retaining the regeneration and differentiation ability of stem cells (Yao et al., 2017; van Dongen et al., 2018; Tonnard et al., 2020).

Tonnard et al. (2013) used an emulsification technique to chylose fat, resulting in “nanofat,” providing a new mode of fat treatment. Nanofat is rich in growth factors and regeneration-related cells with the potential to play a role in cell therapy but is not suitable for filling the soft-tissue volume (Tonnard et al., 2013, 2020; Uyulmaz et al., 2018; Chen et al., 2019; Verpaele et al., 2019; Pallua and Kim, 2020). Yao et al. (2017) also used mechanical shear force to obtain a gelatinous fat that was named stromal vascular fraction (SVF)/extracellular matrix (ECM) gel, which also contained a rich source of regeneration-related cells and ECM, demonstrating the potential for cell therapy (Yao et al., 2017). Both the emulsification and shear force methods involve complete physical approaches without requiring an enzyme.

Accumulating evidence indicates that activating cells through mechanical signals in the extracellular microenvironment can mediate a series of signaling pathways through cytoskeleton rearrangement, thereby altering the proliferation and differentiation abilities of progenitor cells (Nava et al., 2012; Sun et al., 2012; Li Y. et al., 2019; Chen et al., 2020; Zhang et al., 2020). An SVF/ECM gel is a type of Coleman fat obtained using standard Coleman technology following centrifugation and is further mechanically isolated. The mechanically disrupted lipoaspirate product is acquired through minimal manipulation methods, resulting in a tissue SVF (tSVF) (Trivisonno et al., 2019). We hypothesized that mechanical isolation not only results in a simple physical effect of thinning the fat but can also ultimately improve fat retention by affecting the regeneration ability of cells in the SVF/ECM gel, i.e., the application of different shear forces may cause biological changes in related cells in the grafts to ultimately affect the regeneration success. The SVF/ECM gel contains abundant vascular matrix fragments, which have been widely used in cell therapy. However, little is known regarding the specific proportion of and changes in regeneration-related cell subsets in an SVF/ECM gel prepared using mechanical shear force.

In this study, we compared the expression of cell surface antigens and the proportion of cell subsets under different shear forces (Coleman fat without shear force and SVF/ECM gel with mechanical isolation *via* application of shear force) and evaluated the retention rate of fat grafts under these conditions in a mouse model. Our study showed that efficient and stable fat retention may be possible through SVF/ECM gel transplantation without any additional cells, thus achieving ideal capacity filling. Additionally, we found that shear force is positively correlated with viscosity, and the viscosities of Coleman fat and SVM/ECM gel differ. These findings can provide a theoretical basis for the clinical application of autologous fat.

## Materials and Methods

### Fat Sample Acquisition and Processing

**FIGURE 1:**
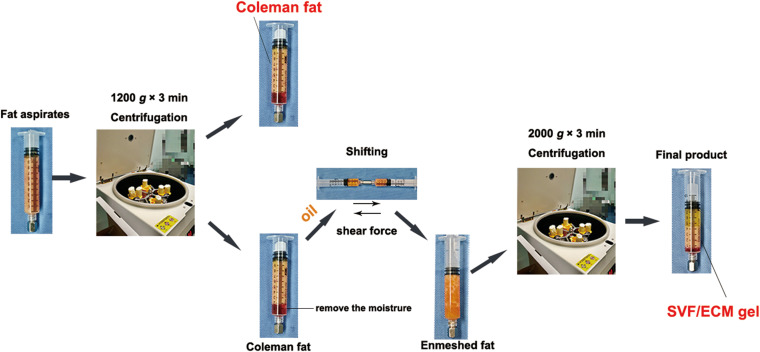
Preparation process of SVF/ECM gel and Coleman fat. Coleman fat was obtained using centrifugation of fat aspirates. The upper oil and fat layers were retained by removing the lower water layer, and the mixture was pushed to form the enmeshed fat. After centrifugation, the SVF/ECM gel was obtained. The whole preparation involves pure physical action, without the use of any foreign additives.

To reduce the influence of potential interference factors, we obtained fat aspirates from the abdomen of seven non-obese women undergoing liposuction without metabolic diseases. The mean (± standard deviation) age of the patients was 34.0 ± 3.6 years with a body mass index of 20.1 ± 1.6 kg/m^2^. This study received approval from the institutional ethics committee of Guangdong Woman and Children Hospital (no. 201801005). All patients provided informed consent for the use of their samples for research purposes. The samples were divided into two groups: Coleman fat group and SVF/ECM gel group. Coleman fat was prepared following the classic Coleman technology method (Coleman, 2006). In brief, a 10-mL syringe was used to connect the tonsil porous liposuction needle with a diameter of 3 mm and a diameter of 1 mm for negative pressure suction, and the obtained liposuction aspirate was further centrifuged at 1,200 × *g* for 3 min. The upper oil and lower water layers were removed, and the fat tissue in the middle was retained without applying shear force; it was collected as the Coleman fat and set as the blank control group. The preparation method of the SVF/ECM gel followed the method described by Yao et al. (2017). In brief, a 10-mL syringe was used to connect the liposuction needle with a diameter of 3 mm and a sharp aperture of 1 mm for negative pressure suction. Liposuction was performed at a suction pressure of –0.75 atm. The obtained liposuction aspirate was further centrifuged at 1,200 × *g* for 3 min to remove the moisture in the lower layer while carefully adding 0.5 mL of oil. Two 10-mL syringes were used with a female-to-female Luer lock connector with an inner diameter of 2.4 mm to push against each other for 1 min at a mutual pushing speed of 10 mL/s. The enmeshed fat was obtained and centrifuged again at 2,000 × *g* for 3 min. After removing water and oil, the middle fat tissue was retained and was collected as the SVF/ECM gel. Figure 1 shows a schematic of the specific operation steps for each group.

### Animal Model Establishment and Experimental Grouping

**FIGURE 2:**
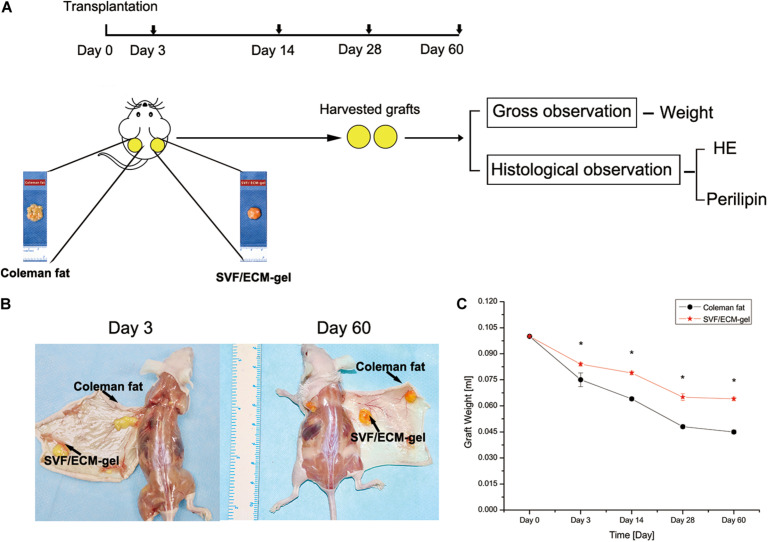
Comparison of the general characteristics and retention rates of fat grafts. **(A)** Experimental design. **(B)** General observation of the two types of fat grafts at 3 and 60 days after transplantation. **(C)** Changes in fat retention rate. *P < 0.001.

This study received approval from the Institutional Animal Care and Use Committee of Guangzhou Medical University according to the rules of the Committee on Animal Research and Ethics (no. GY2018-018). Six- to eight-week-old male specific pathogen-free BALB/C nude mice purchased from Guangdong Medical Experimental Animal Center were selected as the transplant recipients. The breeding and operation of nude mice were conducted in the experimental animal center of Guangzhou Medical University. For the experimental group, human adipose tissue was divided into two groups: Coleman fat group and SVF/ECM gel group. Each fat type was prepared according to the descriptions outlined in Section “Fat Sample Acquisition and Processing” (Figure 1). The nude mice were anesthetized with an intraperitoneal injection of pentobarbital sodium (50 mg/kg). The procedure was performed in 25 nude mice per group, and Coleman fat (0.1 mL) and SVF/ECM gel (0.1 mL) were both transplanted onto the backs of each nude mouse simultaneously to establish a fat transplantation nude mouse model using a 1-mL syringe with a blunt-tipped, 14-gauge infiltration cannula. Therefore, the total number of samples was 50. All nude mice were euthanized, and the grafted fat was harvested after 0, 3, 14, 28, and 60 days (*n* = 5 at each time point) (Figure 2). Grafted samples were fixed in 4% paraformaldehyde for 24 h, then dehydrated, and paraffin-embedded for histological examination.

### General and Histological Observation of Fat Grafts

The general and histological changes in fat samples were observed and recorded at days 3, 14, 28, and 60 after fat transplantation, and the long-term retention rate was evaluated. Five-micrometer-thick specimens were prepared and stained with hematoxylin and eosin (HE). Images were captured with an Olympus BX51 microscope and Olympus Dp71 digital camera for analysis. The following antibodies were used for immunostaining: guinea pig anti-mouse perilipin as the primary antibody (diluted 1:400; Progen, Heidelberg, Germany) and Alexa fluor 647-labeled goat anti-guinea pig immunoglobulin G (diluted 1:2,000; Abcam) as the secondary antibody. The cell nucleus was labeled with DAPI (diluted 1:200; Sigma, St. Louis, MO, United States). All data were collected and analyzed by two observers.

### Calculation of Fluid Shear Force

Since the SVF/ECM gel was obtained through mechanical isolation of Coleman fat, the shear stresses of Coleman fat and the SVF/ECM gel were compared by measuring their respective viscosities to evaluate the effect of different degrees of shear stress on the cell behavior of the grafts. The viscosity of the fluid was measured using a DV-IPRIME Viscometer (Brookfield Engineering Laboratories, Inc., Boulevard, MA, United States). First, the Reynolds coefficient (Re) was calculated to evaluate whether the fluid flow characteristics were laminar or turbulent, using the following formula: Re = 2q/πν R, where q represents the volume flow of the measured medium, ν represents the kinematic viscosity of the medium, and R represents the radius of the adapter pipe connecting the syringe. A Re value < 2,300 represents laminar flow, Re of 2,300–4,000 represents a transition state, and Re > 4,000 indicates turbulent flow. If laminar flow was detected, the shear stress (τ) of the fat sample was further calculated with the formula *τ* = 4 μ Q/π R3, where μ represents dynamic viscosity, calculated as ν × ρ in which ρ is the approximate water density (Banyard et al., 2016).

### Separation of the SVF

Coleman fat and the SVF/ECM gel were prepared from 10-mL fat samples as described above and further processed for obtaining the SVF as described previously (Banyard et al., 2016; Yao et al., 2017). In brief, the samples were washed with phosphate-buffered saline repeatedly to remove the blood. Thereafter, 0.075% collagenase I (Sigma–Aldrich Co., St. Louis, MO, United States) was added to the fat tissue samples and digested in a constant temperature shaker at 37°C for 30 min. The sample was then mixed repeatedly two to three times; neutralized in a complete medium of equal volume high-glucose Dulbecco’s modified Eagle medium, 10% fetal bovine serum, and 1% penicillin-streptomycin (Sigma); and centrifuged at 800 × *g* for 5 min. The cell pellets were then resuspended and filtered through a 100-μm mesh. Red blood cell lysate (Sigma) was added to minimize erythrocyte contamination and incubated at 37°C for 5 min, followed by a final centrifugation at 800 × *g* for 5 min. The aqueous portions were removed after each centrifugation step. The pellets were then resuspended in control media and subjected to staining and analysis.

### Analysis of the Number, Density, and Activity of SVF Cells

Stromal vascular fraction cells in the fresh suspension from the two groups of samples were isolated and counted using fluorescence double staining. Additionally, for apoptosis analysis, the cells were incubated with fluorescein isothiocyanate-conjugated Annexin V (Caltag, Burlingame, CA, United States) at 37°C for 20 min and analyzed directly with propidium iodide (PI) using flow cytometry.

### Phenotype Analysis of SVF Cells

Fluorescence-activated cell sorting was used to analyze the cell surface markers in the SVF cell suspensions using the following monoclonal antibodies: mouse anti-human CD45-PE-Cytm5, CD31-PE-Cytm7, CD34-FITC, CD146-PE, CD13-PE, and CD73-FITC (BD Biosciences). The single-cell suspension was placed in a polystyrene tube on ice. An LSR II (BD Biosciences) flow cytometer was used to detect the cell density and cell surface markers in the SVF suspension.

### Proportion of Cell Subsets in the SVF Cell Suspension

Multiple fluorescence staining was performed in the fresh SVF cell suspensions obtained from Coleman fat and the SVF/ECM gel. The number and proportion of cell subsets in SVF cell suspensions were analyzed through detection of their markers: endothelial progenitor cells (EPCs), CD45^–^CD31^+^CD34^+^CD146^+^; adipose-derived stem cells (ASCs), CD45^–^CD31^–^CD13^+^CD73^+^; transitional cells, CD45^–^CD31^–^CD34^+^CD146^+^; pericytes, CD45^–^CD31^–^CD34^–^ CD146^+^; and supra-adventitial (SA)-adipose stromal cells (ASCs), CD45^–^CD31^–^CD34^+^CD146^–^ (Zimmerlin et al., 2013; Guo et al., 2016).

### Statistical Analysis

Data are expressed as mean ± standard deviation and were analyzed using SPSS 20.0. Independent sample *t*-tests were used to compare the data of the two groups at a single time point. *P* < 0.05 was considered as a statistically significant difference.

## Results

### General Observations and Long-Term Retention of Grafts

At 3, 14, 28, and 60 days after fat transplantation, the retention rate of the SVF/ECM gel graft was significantly higher than that of the Coleman fat graft at all time points (all *P* < 0.001; Figure 2).

### Histological Observation of Grafts

**FIGURE 3:**
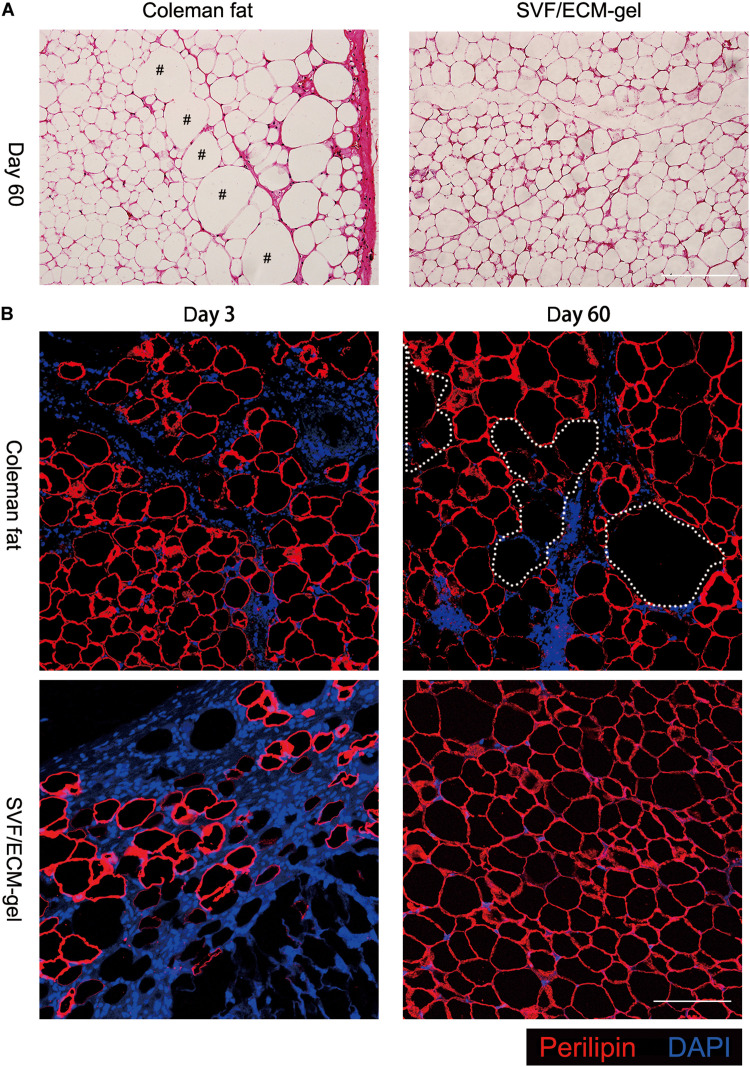
Histological changes in the transplanted fat. **(A)** Hematoxylin and eosin staining on day 60 after transplantation showing that the SVF/ECM gel formed a mature lobular fat structure (right), whereas some oil droplets remained in the Coleman fat grafts (left); scale bars = 200 μm. **(B)** Fluorescence staining showing that 3 days after transplantation, the adipocytes in the SVF/ECM gel grafts were necrotic, and the negative area of perilipin was significantly increased (lower left), compared to the Coleman fat grafts (upper left); 60 days after transplantation, a large number of new adipocytes with positive perilipin staining were found in the SVF/ECM gel grafts (lower right), with many negative areas (upper right) of perilipin in the Coleman fat grafts; scale bars = 100 μm.

Hematoxylin and eosin staining showed that 60 days after transplantation, the SVF/ECM gel formed a mature lobular fat structure, whereas some oil drops remained in the central area of the Coleman fat graft sites (Figure 3A). The adipocytes were labeled using perilipin, which is a protein that coats lipid droplets only in viable adipocytes (Eto et al., 2012; Kato et al., 2014; Doi et al., 2015). Three days after transplantation, compared with those in the Coleman fat grafts, the adipocytes in the SVF/ECM gel grafts were necrotic and the negative area of perilipin significantly increased due to destruction by the applied shear force. Sixty days after transplantation, a large number of new adipocytes with positive perilipin staining were found at the SVF/ECM gel transplant site, and the size and morphology were regular, whereas several negative areas of perilipin were observed at the Coleman fat graft site (Figure 3B).

### Relationship Between Viscosity and Shear Force

**FIGURE 4:**
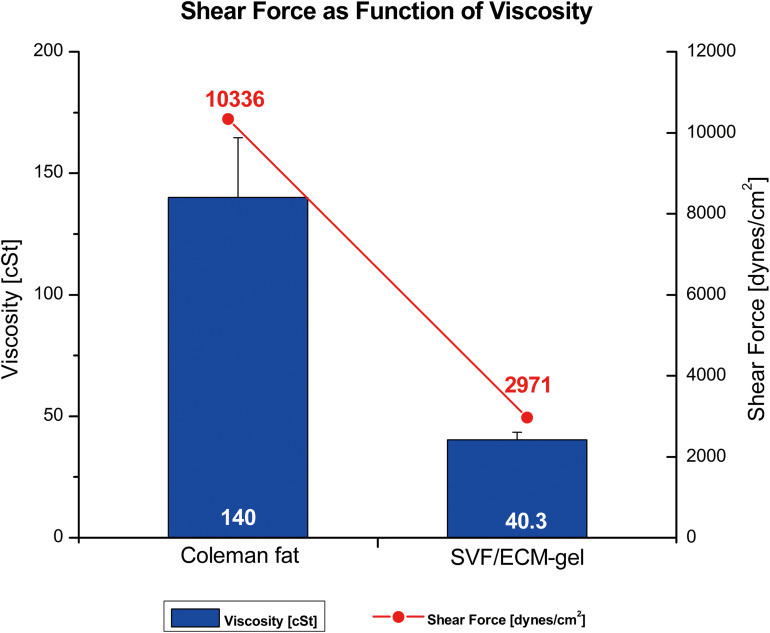
Relationship between shear force (line) and kinematic viscosity (columns) of different fat types. The SVF/ECM gel was obtained by mechanical isolation of the initial Coleman fat through the application of shear force.

The relationship between shear force and kinematic viscosity in the process of mechanical isolation from standard Coleman fat to the SVF/ECM gel was analyzed. The fat was in an enmeshed state before SVF/ECM gel centrifugation; thus, the kinematic viscosity of Coleman fat was much higher than that of the SVF/ECF gel measured under the same connector radius and volume flow of 1.2 mm and 10 mL/s, respectively. The Re values were 65.9 and 18.9 for the SVF/ECF gel and Coleman fat, respectively, indicating laminar flow. The shear force of Coleman fat was also much higher than that of the SVF/ECM gel (Figure 4). These results demonstrated that with the decrease in viscosity, the ability to produce shear force was reduced.

### Number, Density, and Activity of SVF Cells

**FIGURE 5:**
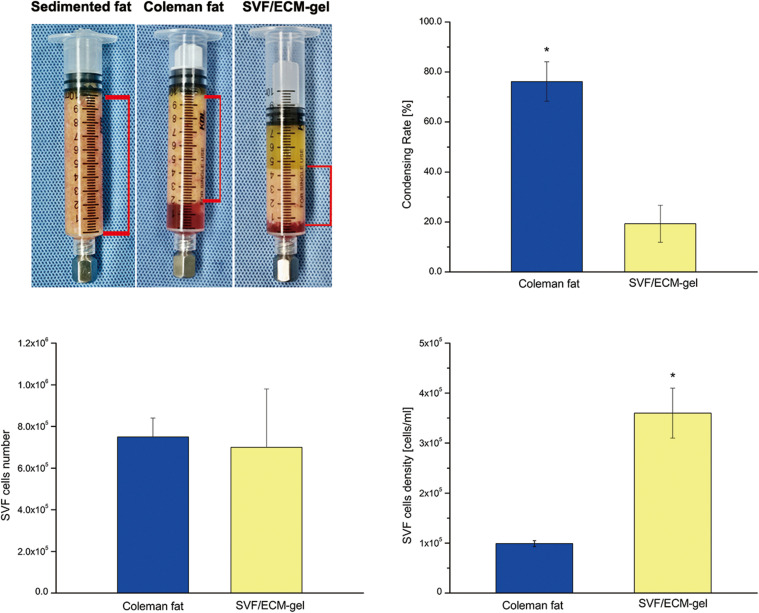
Condensation rate of the fat samples and the total number and density of SVF mixed cells. General observation **(upper left)** and statistical analysis **(upper right)** of the volume changes in the two types of fat samples with the same initial volume during the preparation of Coleman fat and SVF/ECM gel. *P < 0.05.

**FIGURE 6:**
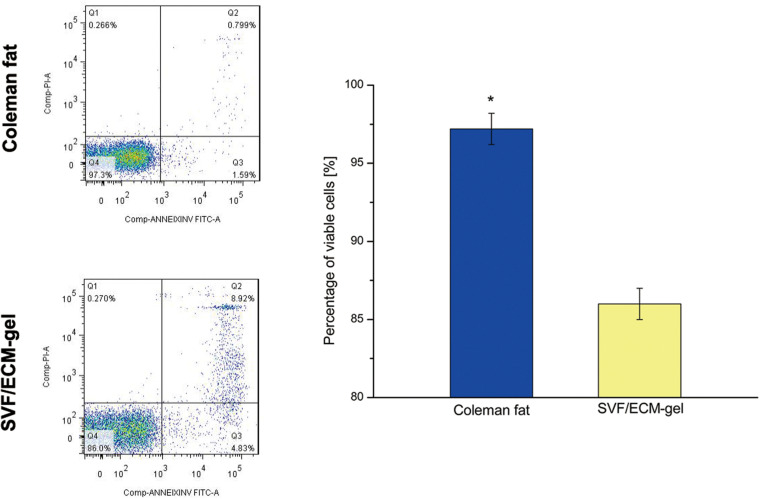
SVF cell activity of the fat grafts. The apoptosis of SVF cells was detected using flow cytometry with Annexin V/PI double staining in Coleman fat and the SVF/ECM gel. *P < 0.05.

As shown in Figure 5, there was no significant difference in the number of SVF cells obtained from the Coleman fat and SVF/ECM gel (*P* = 0.681), and the final volume of the SVF/ECM gel was about one-quarter that of the Coleman fat (*P* < 0.05). In addition, the cell density of the SVF/ECM gel graft increased significantly compared to that of the Coleman fat graft (*P* < 0.05). However, the number of living cells in the Coleman fat graft site was significantly higher than that in the SVF/ECM gel graft site (*P* < 0.05; Figure 6).

### Phenotype Analysis of SVF Cells in Grafts

**FIGURE 7:**
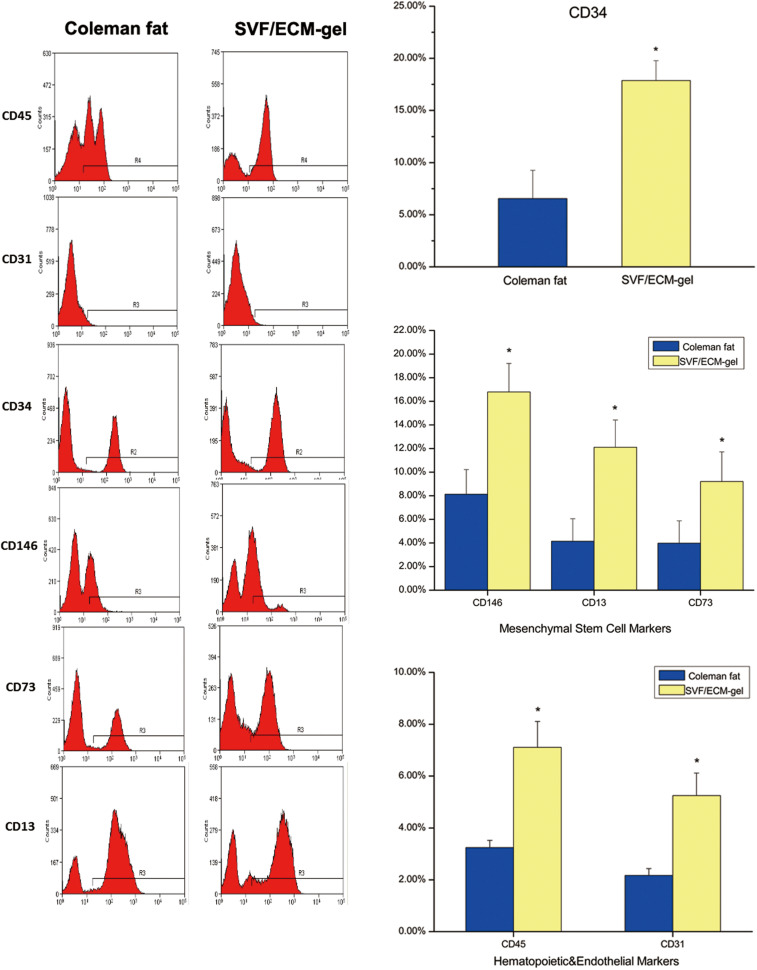
Flow cytometry for cell surface markers of SVF cells from the two fat types. The oscillogram represents the phenotypes of SVF cells isolated from the Coleman fat group **(left)** and SVF/ECM gel group **(right)**. *P < 0.05.

Compared with the Coleman fat grafts, the SVF/ECM gel grafts showed a more than threefold increase in the levels of the general stem cell marker CD34 (*P* < 0.05), mesenchymal stem cell markers, and hematopoietic markers (*P* < 0.05) (Figure 7). Thus, the expression of these markers in the SVF/ECM gel grafts was induced by shear stress, becoming significantly higher than that in the Coleman fat grafts without shear stress.

### Proportion of Cell Subsets in the SVF Cell Suspension

**FIGURE 8:**
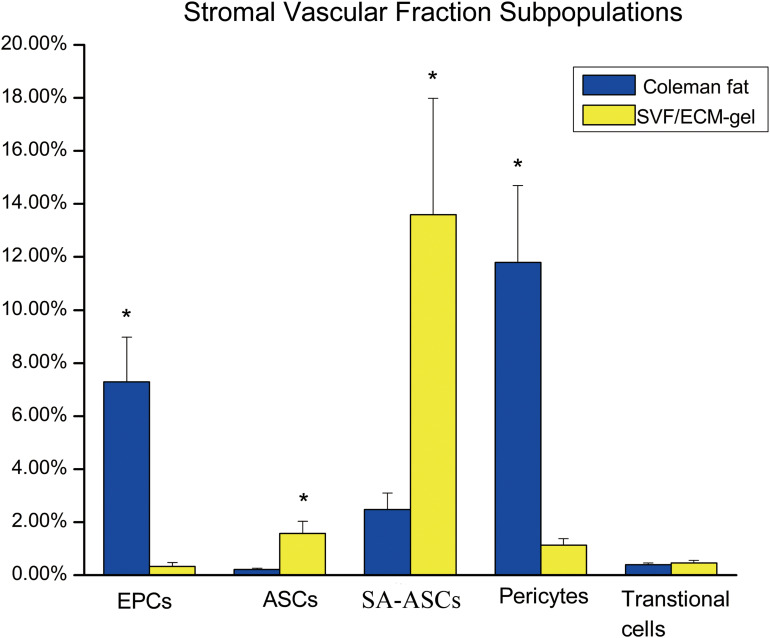
Flow cytometry of the proportions of stem cell subsets in the suspension of SVF cells from the two fat grafts. ASC, adipose-derived stem cell; EPS, endothelial progenitor cell. *P < 0.05.

The proportions of ASCs (CD45^–^CD31^–^CD13^+^CD73^+^) and SA-ASCs (CD45^–^CD31^–^CD34^+^CD146^–^) increased significantly in the SVF/ECM grafts compared to those in the Coleman fat grafts. However, the proportions of EPCs (CD45^–^CD31^+^CD34^+^CD146^+^) and peripheral cells (CD45^–^CD31^–^CD34^–^CD146^+^) decreased significantly in the SVF/ECM grafts compared to those in the Coleman fat grafts. There was no significant difference in the proportion of transitional cells (CD45^–^CD31^–^CD34^+^CD146^+^) between the two graft types (Figure 8).

## Discussion

Development of new strategies to improve the retention rate following fat transplantation has become a research hotspot. The activity of adipocytes is affected by fat preparation and processing methods (Oranges et al., 2019; Sese et al., 2019; Wei et al., 2019). The key factor contributing to the fat retention rate is the extent of retainment of the integrity and activity of the adipocyte structure in the fat graft (Yuan et al., 2015). In recent years, Kato et al. (2014) proposed the three-zone theory of fat transplantation, based on two older theories: the cell survival theory, which states that only one to three layers of mature adipocytes could survive in grafts due to the lack of early blood supply and oxygen content, and the host cell replacement theory, indicating that new adipocytes are formed almost exclusively by the differentiation of ASCs (Billings and May, 1989). Several studies subsequently confirmed this theory of fat regeneration (Doi et al., 2015; Dong et al., 2015; Ye et al., 2017; Tang et al., 2018). Therefore, the key to fat regeneration is to increase the content of SVF cells and ASCs. At present, the cell-assisted fat transplantation technology Lipotransfer involves the addition of regenerated cells (ASCs or SVF cells), which has proven to significantly improve the retention rate of fat grafts in small-scale fat transplantation (Zhang et al., 2016; Zhou et al., 2016; Gontijo-de-Amorim et al., 2017; Li et al., 2017; Cai et al., 2018; Bashir et al., 2019; Hong et al., 2019; Kim et al., 2019). However, this technology is limited based on safety challenges such as enzyme digestion, contamination, and possible promotion of tumor recurrence in the process of regeneration related to the steps of cell separation; these have hindered its clinical use (Fraser et al., 2011; Krumboeck et al., 2013; Orbay et al., 2018; Gebremeskel et al., 2019).

In this study, we improved the technology of SVF/ECM gel preparation introduced by Yao et al. (2017), in which mechanical shear force was used to prepare the SVF/ECM gel, which is also commonly described as tSVF (Trivisonno et al., 2019). With no filtering technique, more ECM is saved, leading to an increase in the relative numbers of regenerative cells such as ASCs, pericytes, and SA-ASCs that attached around vessels embedded in the ECM compared to the results obtained in the original study on this technique (Yao et al., 2017). Moreover, compared to Coleman fat, there was no significant difference in the number of mixed cells isolated from the SVF/ECM. Interestingly, we found a higher number of living cells in the Coleman fat grafts than in the SVF/ECM gel. Since the total number of cells was the same in the SVF/ECM gel and Coleman fat, given that the same initial volume of fat samples was used, and the lowest layer of blood was discarded after centrifugation in both cases, the mechanical isolation step involved in SVF/ECM gel processing likely did not affect the live cell number. However, since the volume of mature adipocytes is much larger than that of other cells, we suspect that mechanical isolation mainly destroys the cell membrane of mature adipocytes, leading to their death. Therefore, the Coleman fat without mechanical isolation had a relatively higher number of total living cells.

Accurately assessing biological changes in cells subject to a mechanical isolation technique is essential. Based on the findings of Zimmerlin et al. (2013) and Banyard et al. (2016), we used a more precise combination of phenotypic markers to identify the proportion of cell subsets among the SVF cells. We used flow cytometry to analyze the expression of each cell surface marker and the change in the proportion of each cell subpopulation in the improved SVF/ECM gel. Although the total number of living cells in the Coleman fat was significantly higher than that in the SVF/ECM gel, the proportions of cells expressing phenotypic (CD34, CD13, CD73, and CD146) and hematopoietic (CD45 and CD31) markers of mesenchymal stem cells were significantly increased in the SVF/ECM gel. This result is consistent with the findings of Banyard et al. (2016). It is likely that the mechanical shear stress liberated many of the SVF cells from the adipose matrix inherent in fat. We found that the relative proportions of ASCs in the unfiltered fat were significantly increased, which is consistent with the results of Yao et al. (2017) and Lombardo and Tamburino (2019). Therefore, the surviving cell population with greater regenerative capacity may account for the favorable results of transplantation in the SVF/ECM gel grafts. Interestingly, the decrease in the proportion of EPCs contrasts with the results of Yao et al. (2017), which may indicate their inaccurate recognition of endothelial cells based only on the CD45^–^CD34^+^CD31^+^ phenotype. We added CD146 as a marker to labeled EPCs to better demonstrate the capacity for neovascularization. In addition, the decrease in the relative proportion of EPCs is likely to be influenced by the increase in the relative proportion of other cells such as mesenchymal stem cells and SA-ASCs.

In contrast to other fat preparation methods, enzyme digestion and filtration are not required for the preparation of SVF/ECM gel. Mechanical shear force alone can destroy mature adipocytes, and oil drops are removed during centrifugation, thus reducing the potential inflammatory reaction in the fat grafts and possible postoperative complications. In addition, we found that the relative proportion of cell subpopulations of the SVF mixed cell group in the fat increased under the shear force. These two findings indicate the potential advantages of the clinical transplantation of SVF/ECM gel compared to Coleman fat. The primary limitation of our study is that our results were all based on image observations, without in-depth molecular-level research. Specifically, the signaling pathways through which mechanical isolation affects the ability of cell proliferation and differentiation along with the relevant key proteins influenced by this process remain unclear. Therefore, the mechanism underlying the observed effects warrants further investigations.

## Conclusion

Mechanical isolation is not an inert process and is more complex than the simple decomposition and refinement of fat tissue for transplantation. In particular, the application of shear force could enhance the pluripotency of cells, and this phenotypic advantage may improve fat retention *in vivo*. Importantly, this study demonstrates the direct biological effects of a physical transformation process with clinical application potential to benefit patients. These effects can help achieve efficient and stable volume filling, while providing an enriched source of regeneration-related cells for cell therapy, thereby advancing the field of clinical tissue repair and reconstruction.

## Data Availability Statement

The original contributions presented in the study are included in the article/supplementary material. Further inquiries can be directed to the corresponding author.

## Ethics Statement

The studies involving human participants were reviewed and approved by the Ethics Committee of Guangdong Woman and Children Hospital. The patients/participants provided their written informed consent to participate in this study. The animal study was reviewed and approved by the Ethics Committee of Guangdong Woman and Children Hospital.

## Author Contributions

YY independently performed the work under the guidance of JJ. JZ and MT provided technical support during tissue slice observation. KH performed the statistical analysis. All authors contributed to the article and approved the submitted version.

## Conflict of Interest

The authors declare that the research was conducted in the absence of any commercial or financial relationships that could be construed as a potential conflict of interest.
